# The putatively high‐altitude adaptation of macaque monkeys: Evidence from the fecal metabolome and gut microbiome

**DOI:** 10.1111/eva.13595

**Published:** 2023-09-25

**Authors:** Dayong Li, Wancai Xia, Xinyuan Cui, Mei Zhao, Kai Huang, Xueyu Wang, Jian Shen, Hua Chen, Lifeng Zhu

**Affiliations:** ^1^ Key Laboratory of Southwest China Wildlife Resources Conservation (Ministry of Education) China West Normal University Nanchong China; ^2^ Key Laboratory of Conservation Biology of *Rhinopithecus roxellana* (Department of Education of Sichuan Province) China West Normal University Nanchong China; ^3^ College of Life Science Nanjing Normal University Nanjing China; ^4^ Huadian Energy Co., Ltd. Tibet China; ^5^ Mingke Biotechnology Hangzhou China; ^6^ School of Medicine & Holistic Integrative Medicine Nanjing University of Chinese Medicine Nanjing China

**Keywords:** adaptation, fecal color, gut microbiome, high altitude, primates, spatial–temporal changes

## Abstract

Animals living in high‐altitude environments, such as the Tibetan Plateau, must face harsh environmental conditions (e.g., hypoxia, cold, and strong UV radiation). These animals' physiological adaptations (e.g., increased red cell production and turnover rate) might also be associated with the gut microbial response. Bilirubin is a component of red blood cell turnover or destruction and is excreted into the intestine and reduced to urobilinoids and/or urobilinogen by gut bacteria. Here, we found that the feces of macaques living in high‐altitude regions look significantly browner (with a high concentration of stercobilin, a component from urobilinoids) than those living in low‐altitude regions. We also found that gut microbes involved in urobilinogen reduction (e.g., beta‐glucuronidase) were enriched in the high‐altitude mammal population compared to the low‐altitude population. Moreover, the spatial–temporal change in gut microbial function was more profound in the low‐altitude macaques than in the high‐altitude population, which might be attributed to profound changes in food resources in the low‐altitude regions. Therefore, we conclude that a high‐altitude environment's stress influences living animals and their symbiotic microbiota.

## 1 INTRODUCTION

High‐altitude environments are the physiological challenges to many animals (Wu et al., 2020). For example, the Tibetan Plateau is the largest and highest plateau in the world and has extremely harsh environmental conditions, such as hypoxia and cold and intense UV radiation (Qiu et al., 2012; Storz & Cheviron, 2021; Wu et al., 2020). Genetic adaptation (e.g., genes involved in the oxygen metabolism) to life on the Tibetan Plateau has been found in several typical mammal genomes, including the yak (*Bos grunniens*) (Qiu et al., 2012), the Tibetan sheep (Wei et al., 2016), the Tibetan antelope (*Pantholops hodgsonii*) (Ge et al., 2013), and the Tibetan horses (Liu et al., 2019).

Mammals and humans living in high‐altitude regions can increase red blood cell production by responding to the lower oxygen saturation in the blood (Beall, 2007; Ding et al., 2014; Hahn & Gore, 2001; Mairbäurl, 1994; Windsor & Rodway, 2007; Yang et al., 2017, 2021), along with an increase in red blood cell turnover or destruction to regulate the red cell mass under hypoxia (Arias & Arias, 2017; Mairbäurl, 1994; Rice & Alfrey, 2005; Risso et al., 2007; Trudel et al., 2022). Moreover, one of the main components of red blood cell turnover or destruction is bilirubin, which is then secreted into the intestine (Fevery, 2008; Hamoud et al., 2018; Wang et al., 2006). Some gut microbes (e.g., *Clostridium ramosum*, *Clostridium perfringens*, *Clostridium difficile*, and *Bacteroides fragilis*) can reduce bilirubin to urobilinoids and/or urobilinogen (Fahmy et al., 1972; Koníčková et al., 2012; Vítek et al., 2005; Vítek & Tiribelli, 2020; Yamamoto et al., 2021). Urobilinogen is then either reduced to stercobilin (the predominant pigment in feces) or reabsorbed into hepatic portal circulation (Fevery, 2008; Hamoud et al., 2018; Wang et al., 2006). The kidney takes up urobilinogens, which are then oxidized to urobilin (Fevery, 2008; Hamoud et al., 2018; Wang et al., 2006). Thus, here, two basic but unexplored questions were raised: (1) Do the feces of mammals living in high‐altitude regions look browner (with a relatively high concentration of stercobilin) than those of the same mammal species living in low‐altitude regions? (2) Compared to low‐altitude populations, are gut microbes involved in urobilinogen reduction enriched in high‐altitude mammal populations?

**FIGURE 1:**
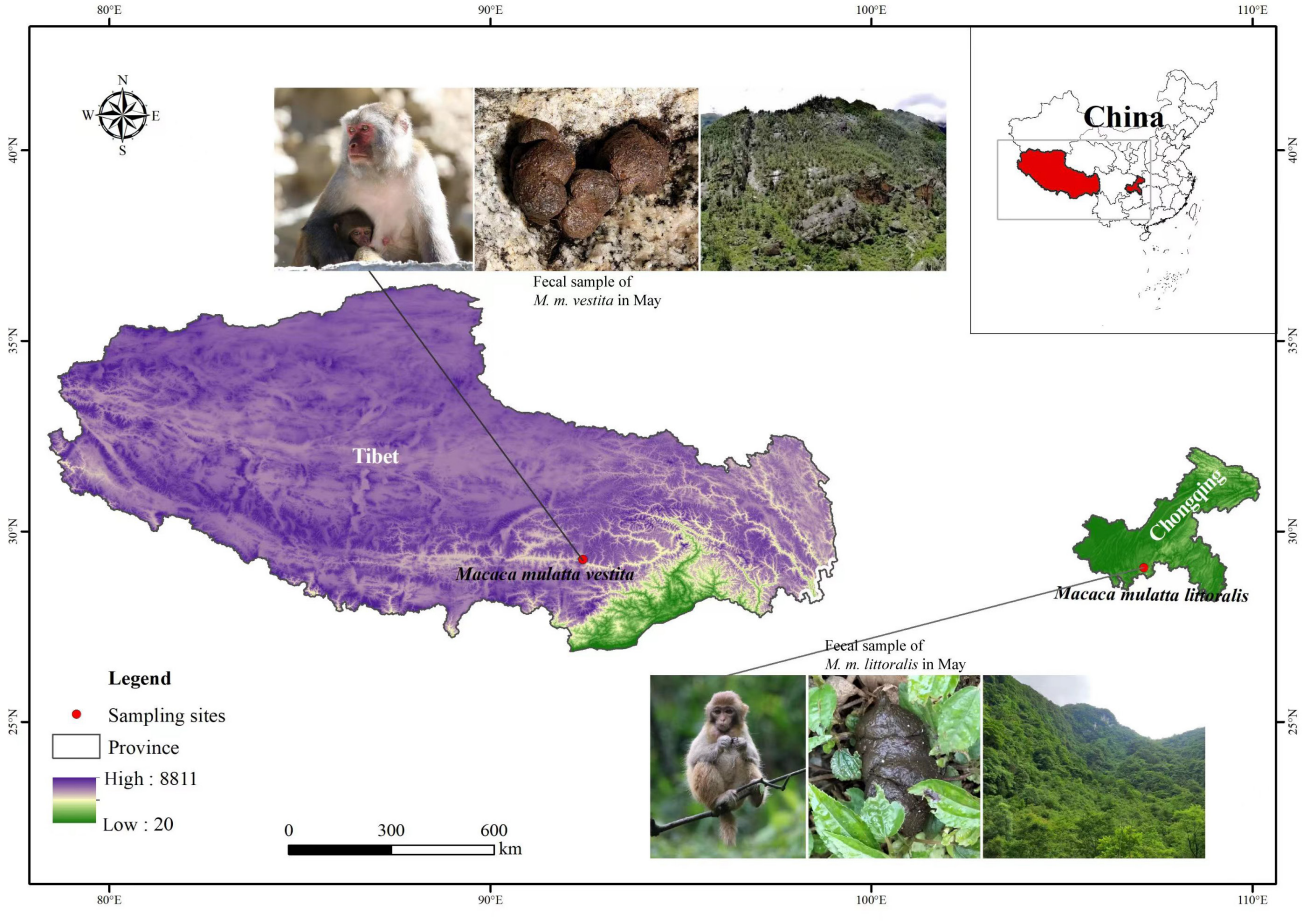
The study sites for the low‐ and high‐altitude macaque populations.

*Macaca mulatta vestita* is a rhesus macaque subspecies with the largest body (51–63 cm) and the highest survival altitude (above 3000 m above sea level), and it is main geographical distribution in East and South Tibet and Northwest Yunnan in China (Jiang et al., 1991). *M. m. vestita* inhabits high mountain forests and stone mountain gorges, with a diet based on buds, leaves, fruits, and seeds of alpine vegetation. *Macaca mulatta littoralis* monkeys live in evergreen broadleaf forests a few hundred meters above sea level, and their foods contain the parts of evergreen higher plants (Jiang et al., 1991). Therefore, the wild macaque subspecies is a good model for exploring the putative changes in the gut microbiome of macaque monkeys living in different altitudes. Multi‐omics methods are now widely used in animal and human gut microbiome studies (Daliri et al., 2021; Milani et al., 2020; Zhao et al., 2022). Thus, in this study, we used multi‐omics strategies on the feces of these two wild macaque subspecies living at different altitudes (Figure 1) to test two simple hypotheses: (1) The concentration of stercobilin in the feces of macaques living in high‐altitude regions is higher than that in the feces of macaques living in low‐altitude regions. (2) Gut microbes involved in urobilinogen reduction are enriched in the high‐altitude macaque population compared to the low‐altitude population. Furthermore, (3) the difference in the food composition between high‐altitude and low‐altitude macaque monkey populations will also influence the gut microbiome.

## 2 MATERIALS AND METHODS

### 2.1 Study site and subject

This study was carried out in Jiacha Gorge on the Yarlung Zangbo River, Tibet, China (92°11′–92°36′ E, 29°7′–29°20′ N), Jinfoshan National Nature Reserve, Chongqing Municipality, China (107°00′–107°22′ E, 28°50′–29°20′ N). Jiacha Gorge is characterized by a plateau climate, where temperature and precipitation are strongly seasonal. The gorge consists of deciduous broad‐leaved forests, mixed coniferous and broad‐leaved forests, and alpine scrub between 3200 and 4752 m above sea level (Figure 1). Jinfoshan National Nature Reserve is located in the subtropical zone, where the vegetation is mainly evergreen broad‐leaf forest (Figure 1). The altitude at the site ranges between 580 and 2251 m. *Macaca mulatta vestita* (MMV) monkeys live at the highest survival altitude (above 3000 meters above sea level), and their main geographical distribution is in East and South Tibet and Northwest Yunnan in China (Jiang et al., 1991). *Macaca mulatta littoralis* (MML) monkeys live in evergreen broadleaf forests a few hundred meters above sea level, and their foods contain the parts of evergreen higher plants (Jiang et al., 1991).

### 2.2 Fecal sample collection

We collected adult fecal samples of two subspecies of rhesus macaques in May 2020 and August 2020. We collected 19 fecal samples (May) and 10 fecal samples (August) of *M. m. vestita* (MMV) from Jiacha Gorge, while we collected 20 fecal samples (May) and 10 fecal samples (August) of *M. m. littoralis* (MML) from Jinfoshan National Nature Reserve. Because these animals are wild populations, we cannot yet identify all the individuals. To avoid repeatedly collecting samples from the same individual, we chose monkey groups inhabiting the two study sites. We shortened the sampling time (fresh fecal sampling is generally completed within 2 h) because these animals usually do not egest two or more times within a short period. We closely observed the monkey groups (for approximately 30 min) to wait for them to egest feces. Fresh fecal samples were collected into sterile centrifuge tubes, sealed, labeled, and retained in mobile dry‐ice buckets until being taken to the laboratory for final storage at −80°C.

### 2.3 Food composition method

We collected food composition data for the two macaque subspecies via scan sampling in May 2020 and August 2020. The method was similar to that used in our previous study (Xia, Zhao, et al., 2022). We obtained 1226 feeding records of *M. m. vestita* (680 in May and 546 in August) and 957 feeding records of *M. m. littoralis* (639 in May and 318 in August).

### 2.4 16S rRNA gene sequencing and analysis

The method was similar to our previous study (Xia et al., 2022). Total DNA was extracted from the 59 fresh fecal samples using the QIAamp DNA Stool Mini Kit (Qiagen). PCR amplification of the bacterial 16S rRNA V3–V4 region was carried out in a 25 μL reaction using universal primer pairs (343F: 5′‐TACGGRAGGCAGCAG‐3′; 798R: 5′‐AGGGTATCTAATCCT‐3′) (Nossa et al., 2010). Sequencing was conducted on an Illumina MiSeq (Illumina et al.; OE Biotech Company). Paired‐end reads were preprocessed using Trimmomatic software (Bolger et al., 2014) and then assembled using FLASH software (Reyon et al., 2012). After trimming, the clean paired‐end reads were further denoised using DADA2 (Callahan et al., 2016) in QIIME2 (Bolyen et al., 2019), following which a table of ASVs and representative sequences was obtained. All representative 16S rRNA gene sequences were annotated and blasted against the SILVA reference database (version 132) (Quast et al., 2012) of the relative abundance levels of ASVs with taxonomic information was obtained in this manner. Samples with different sequencing depths were normalized by rarefying the ASV table to the minimum number (58,380) of sequences observed in all 59 samples. LEfSe was used to determine significant differences in the abundance of gut microbiomes among low‐ and high‐altitude samples in each sampling season (Segata et al., 2011).

### 2.5 Metagenomic sequencing and binning analysis

Six randomly selected samples from each group were used for metagenomic sequencing on the Illumina HiSeq‐PE150 platform. The raw data for each metagenome were approximately 10 G, and approximately 150 G raw reads were obtained. The raw data was treated using the same methods as those used in our previous study (Li et al., 2023; Xia et al., 2022). Cutadapt was used to filter the raw reads based on default parameters (Martin, 2011). Host contamination was deleted based on the rhesus macaque genome (GCF_003339765.1). MEGAHIT was then used to assemble the clean reads (Li et al., 2015). All coding regions of the contigs were predicted using MetaGeneMark (Zhu et al., 2010) and clustered using CD‐HIT (Fu et al., 2012), yielding the unigenes. Transcripts per million (TPM) values were used to estimate the abundance of unigenes according to the number of aligned reads using bowtie2 (Langmead & Salzberg, 2012). Diamond (Buchfink et al., 2015) was used to align the unigenes against the NCBI micro‐Non‐Redundant (NR) database (including bacteria, fungi, archaea, and viruses). We only retained the unigenes belonging to bacteria for the following analysis. Subsequently, taxonomic information on these bacterial unigenes was obtained. Functional annotation of the unigenes was performed against the KEGG and CAZy databases (Cantarel et al., 2009).

The putative genes in the target KEGG pathway were blasted against the NCBI NR database using diamond (Buchfink et al., 2015). Subsequently, we determined the putative microbiome sources of these KEGG pathways within each metagenome. LEfSe was used to determine the significant difference in the abundance of KEGG pathways between low‐ and high‐altitude samples in each sampling season (Segata et al., 2011). We blasted the identified genes against the Antibiotic Resistance Genes Database using SARG2.0 with default parameters (Yin et al., 2018). We then obtained the putative ARG assignments for these genes per metagenome. We applied the PCoA ordination and adonis test in the vegan package on the basis of the Bray–Curtis distance matrices among the groups (Beals, 1984) using bacterial species or functional composition tables. We used the Burrows–Wheeler Aligner algorithm (Li, 2013), Samtools (Li et al., 2009), and MetaBAT2 (Kang et al., 2019) to obtain the contigs for each bin. These bins were searched against the KEGG pathway database using diamond (Buchfink et al., 2015). To determine whether groups of the macaque monkey's fecal samples are significantly different, we used the Adonis permutation‐based statistical test in Vegan (Anderson, 2001; Dixon, 2003). The function partitions sums of squares of a multivariate data set at 999 permutations with the adjusted *p*‐value for multiple comparisons.

### 2.6 Metabolome analysis

Fifty‐nine fecal samples were used in the metabolome experiment. The metabolome experiment was conducted by Shanghai Lu‐Ming Biotech Co., Ltd. The protocol can be found in Appendix S1. The original LC–MS data were processed by software ProgenesisQIV2.3 (Nonlinear, Dynamics) for baseline filtering, peak identification, integral, retention time correction, peak alignment, and normalization. Main parameters of 5 ppm precursor tolerance, 10 ppm product tolerance, and 5% production threshold were applied. Compound identifications were based on precise mass‐to‐charge ratio(*M*/*z*), secondary fragments, and isotopic distribution using the Human Metabolome Database (HMDB), Lipidmaps (V2.3), Metlin, EMDB, PMDB, KEGG, and self‐built databases to do qualitative analysis. The extracted data were further processed by removing any peaks with a missing value in more than 50% in groups. Compounds with resulting scores below 36 (out of 60) points were also deemed to be inaccurate and removed. Then, we gained the metabolite composition for each database. Then, we used MetaboSignal (Rodriguez‐Martinez et al., 2017) to generate the enrichment information in the KEGG pathways based on our KEGG metabolite composition A heatmap of concentrations with z‐score conversion was generated for the enriched metabolites using a heatmap in R (Kolde & Kolde, 2018).

### 2.7 The color of the feces

The feces photos of *M. m. vestita* and *M. m. littoralis* were adjusted to the same size with achieve balanced brightness. We selected 10 parts of these photos for color analysis vas Python 3.8. This analysis showed that the significant difference in the mean RGB (Red Green Blue color channel) values of two feces colors (Wilcoxon Test: *Z* = −2.934, *p* < 0.01).

## 3 RESULTS AND DISCUSSION

### 3.1 Significantly high concentration of stercobilin in the feces of macaques living at high altitudes

**FIGURE 2:**
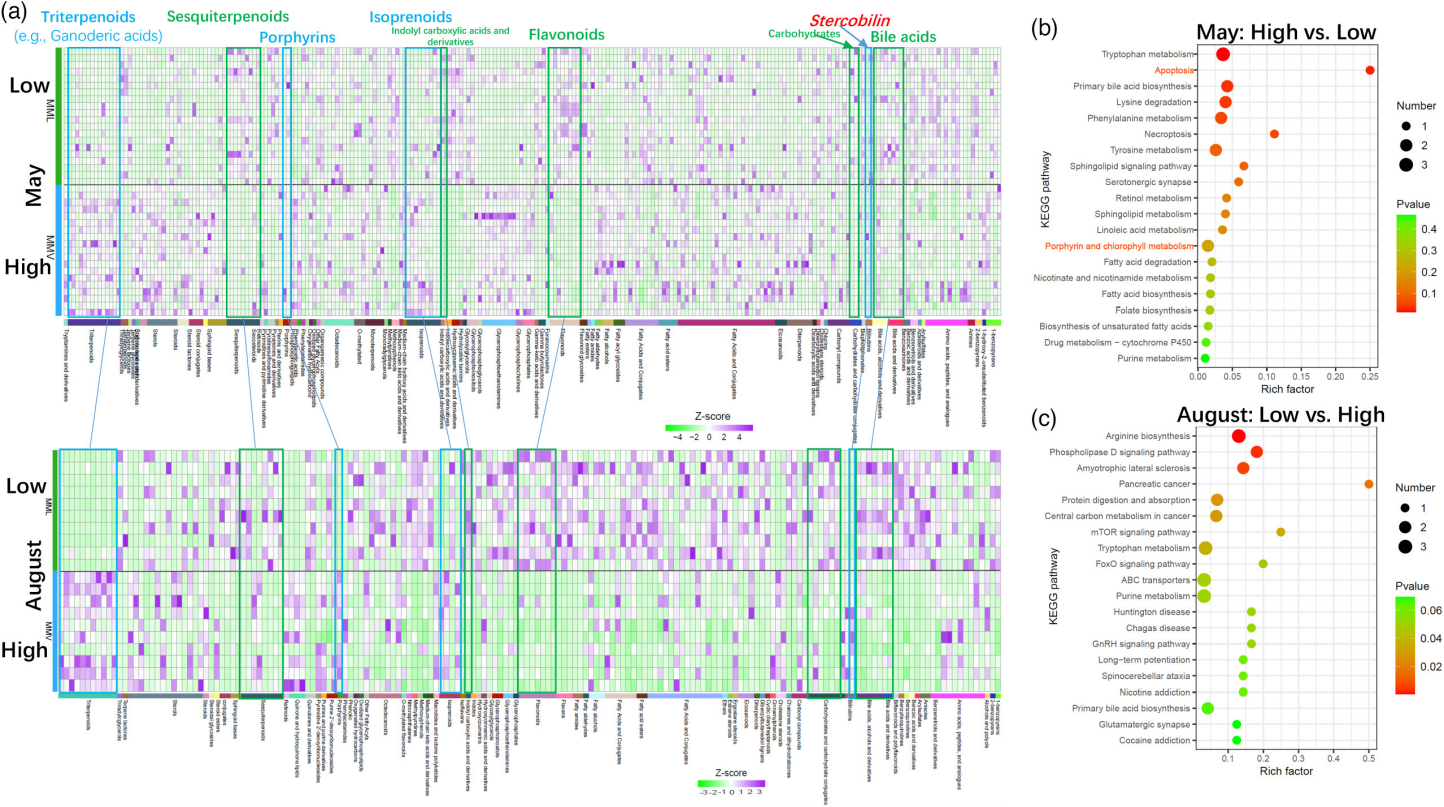
The difference in the concentration of the fecal metabolites between the low‐ and high‐altitude samples. (a) The consistently significant changes in the concentration of the fecal metabolites between the low‐ and high‐altitude samples in each sampling season. Heatmap with z score conversion of the concentration. (b) The enrichment (May: high‐ vs. low‐altitude populations) of the KEGG pathways using the metabolome in the high‐altitude population. (c) The KEGG pathways' enrichment (August: low‐ vs. high‐ populations) using the metabolome in the low‐altitude population. The values in the legend are the *p* values. Aug, August; MML, *Macaca mulatta littoralis* living in the low‐altitude region; MMV, *Macaca mulatta vestita* living in the high‐altitude region.

Here, we found that the feces of macaque monkeys living in the high‐altitude region were dark brown compared to those of low‐altitude monkeys (Figure 1 and Figure S1, Wilcoxon Test: *Z* = −2.934, *p* < 0.01). The metabolome data from 59 fresh fecal samples across two sampling seasons (May and August: Table S1) confirmed this finding, that is, the concentration of stercobilin (belonging to the bilirubin subclass) in the feces of macaques living in the high‐altitude region was significantly higher than that of low‐altitude monkeys in May and August (Figure 2a). Another feature of the high‐altitude samples was that the concentrations of some metabolites belonging to the porphyrin family were also significantly higher than those in low‐altitude monkeys in May and August (Figure 2a). KEGG enrichment analysis indicated the top 20 upregulated pathways, including porphyrin, chlorophyll metabolism, and apoptosis (Figure 2b,c). The KEGG database classifies the bilirubin pathway under porphyrin and chlorophyll metabolism. Hypoxia induces apoptosis in high‐altitude environments (Kosanovic et al., 2019; Li et al., 2017; Wu et al., 2020). A third feature of the high‐altitude samples was upregulated apoptosis. Thus, we speculated that increased red blood cell production and turnover led to increased bilirubin levels. After excretion and reduction in the body, one of the final metabolites in the feces was stercobilin as the predominant pigment. Therefore, comparative analysis of the metabolome supported the first hypothesis, which putatively reflects the effects of high‐altitude adaptation on macaque physiology.

### 3.2 The enriched gut microbiota involved in urobilinogen metabolism in the high‐altitude samples

**FIGURE 3:**
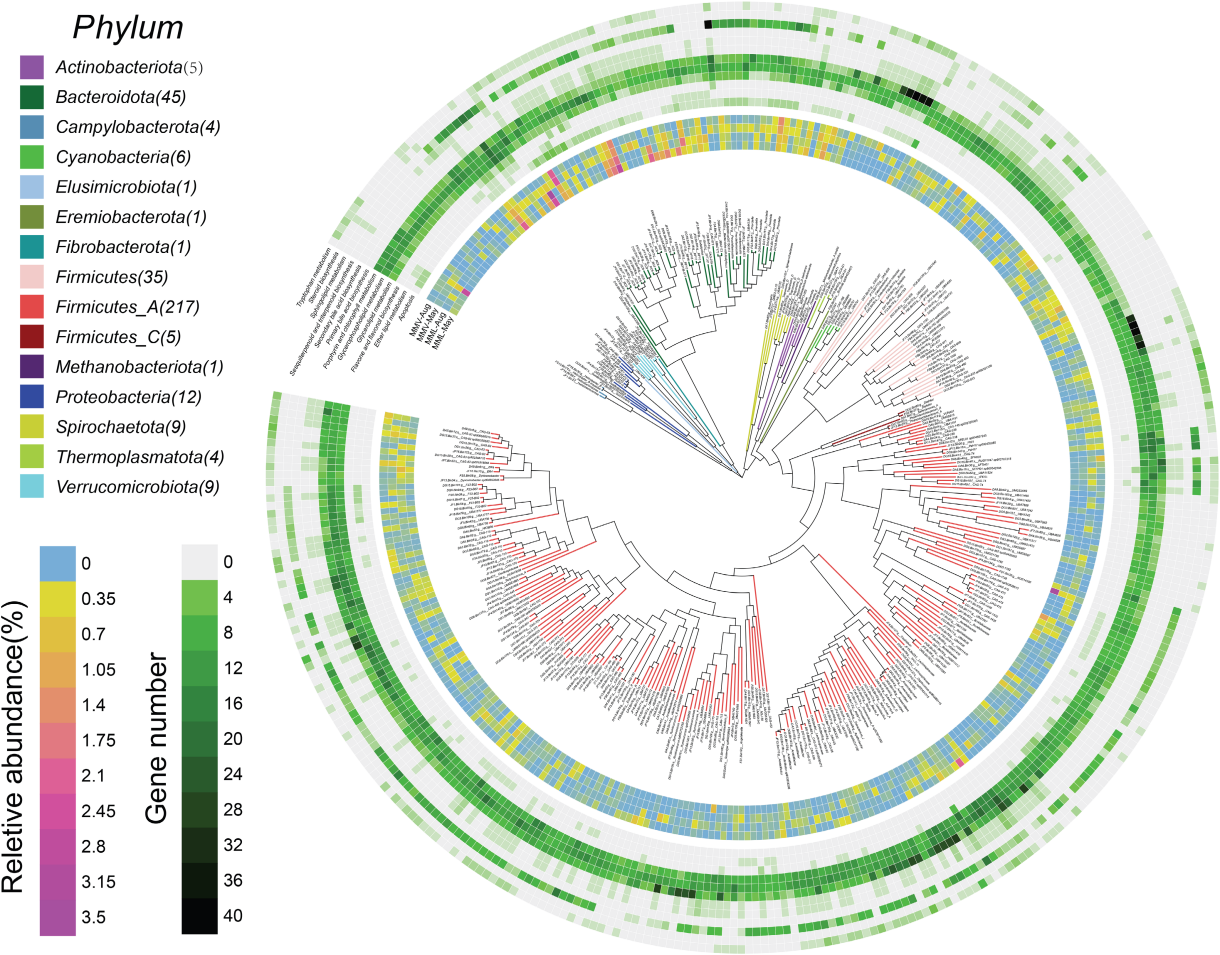
The distribution of the 355 nonredundant MAGs (metagenomic assembled genomes) using 24 metagenomes. Phylogenetic analysis (using PhyloPhlAn [Segata et al., 2013]) of these 355 MAGs (coverage >80%, contamination rate < 10%). The panels in the center circle display the maximum‐likelihood trees created using the MAGs. The outer circle heatmap shows the relative abundance of each bin (MAG) in each macaque group or the gene numbers in each specific pathway. Aug, August; MML, *Macaca mulatta littoralis* living in the low‐altitude region; MMV, *Macaca mulatta vestita* living in the high‐altitude region.

**FIGURE 4:**
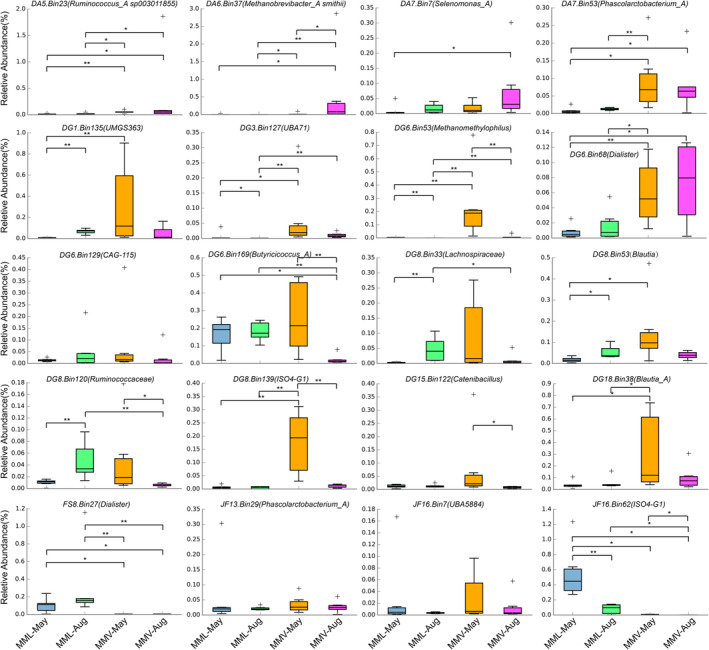
The relative abundance of 20 MAGs harboring a high number of genes encoding putative enzymes involved in porphyrin and chlorophyll metabolism among each macaque group. Pairwise comparisons were based on the Kruskal–Wallis test (Bonferroni *p*‐value). Aug, August; MML, *Macaca mulatta littoralis* living in the low‐altitude region; MMV, *Macaca mulatta vestita* living in the high‐altitude region.

**FIGURE 5:**
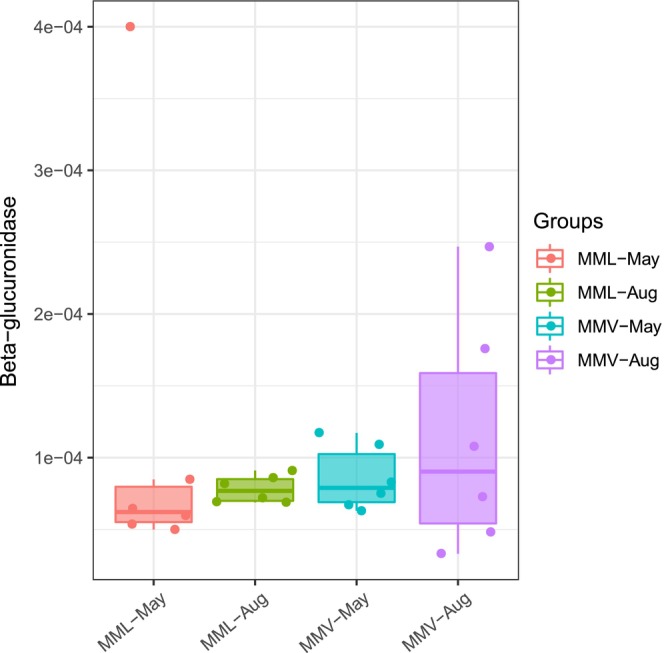
The relative abundance of genes coding for putative beta‐glucuronidase [EC 3.2.1.31], involved in reducing bilirubin beta‐diglucuronide to d‐urobilinogen using 24 metagenomes. Pairwise comparisons were based on the Kruskal–Wallis test (Bonferroni *p*‐value).

Bilirubin is excreted into the intestine and reduced to urobilinoids and/or urobilinogens by gut bacteria (Fahmy et al., 1972; Koníčková et al., 2012; Vítek et al., 2005; Vítek & Tiribelli, 2020; Yamamoto et al., 2021). Interestingly, we observed the enrichment of gut microbes involved in urobilinogen metabolism in the high‐altitude samples (Figure 3). We obtained 355 high‐quality metagenome‐assembled genomes (MAGs) from 24 metagenomes across two sampling seasons (May and August: Table S1) and found that approximately 20 MAGs (putatively belonging to *Ruminococcus*, *Methanobrevibacter*, *Selenomonas*, *Phascolarctobacterium*, *Methanomethylophilus*, *Dialister*, *Blautia*, and *Lachnospiraceae*) harbored a high number of genes encoding the putative enzymes involved in porphyrin and chlorophyll metabolism (Figures 3 and 4). The relative abundance of most of these 20 MAGs was higher in the high‐altitude samples than in the low‐altitude samples within the same sampling season (Figure 4). Then, we focused on the genes for bilirubin reduction and found the relative abundance of genes coding for putative beta‐glucuronidase [EC 3.2.1.31], involved in the reduction of bilirubin beta‐diglucuronide to D‐urobilinogen, were higher in the high‐altitude metagenomes than that in the low‐altitude metagenomes (Figure 5).

**FIGURE 6:**
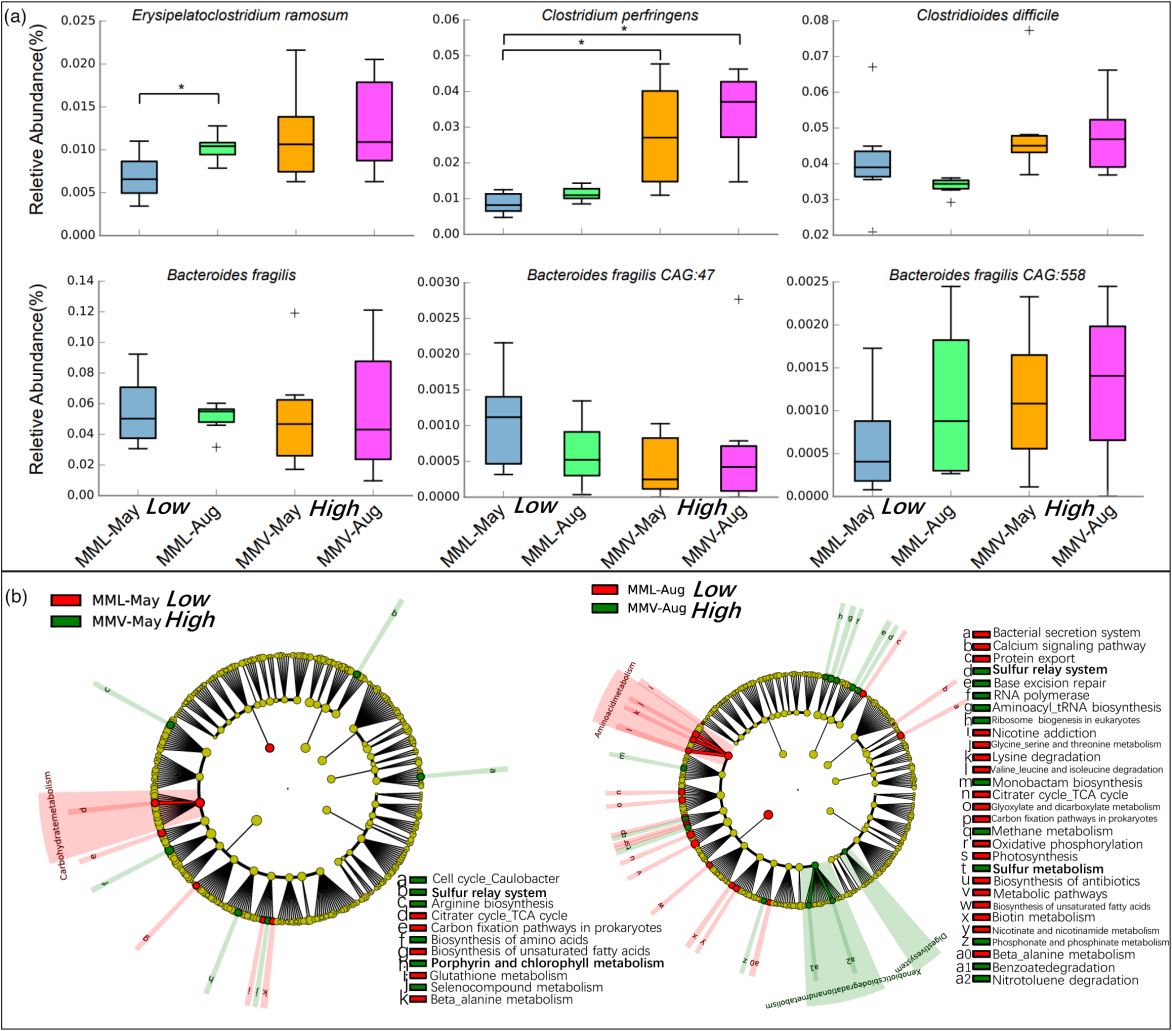
Putative gut microbial response to the high‐altitude environment using 24 metagenomes. (a) The relative abundance of four gut microbial species involved in bilirubin metabolism within each macaque group. Pairwise comparisons were based on the Kruskal–Wallis test (Bonferroni *p*‐value). (b) Linear discriminant analysis effect size was used to determine the significant difference (*p* < 0.05) in the relative abundance of KEGG pathways between low‐ and high‐altitude macaque populations in each sampling season. Aug, August; MML, *Macaca mulatta littoralis* living in the low‐altitude region; MMV, *Macaca mulatta vestita* living in the high‐altitude region.

Moreover, four bacterial species (*Clostridium ramosum*, *Clostridium perfringens*, *Clostridium difficile*, and *Bacteroides fragilis*) can reduce bilirubin to urobilinoids and/or urobilinogen (Fahmy et al., 1972; Koníčková et al., 2012; Vítek et al., 2005; Vítek & Tiribelli, 2020; Yamamoto et al., 2021). The relative abundance of most of these four species based on taxon annotation using metagenomes was higher in the high‐altitude samples than in the low‐altitude samples within the same sampling season. However, only the relative abundance of *C. perfringens* was significantly higher in high‐altitude samples (Figure 6a). The 16S data further confirmed that the relative abundance of some bacteria (e.g., Clostridiaceae, Ruminococcaceae, and Lachnospiraceae) involved in porphyrin and chlorophyll metabolism was higher in the high‐altitude samples than in the low‐altitude samples, especially in May (Figure S2).

**FIGURE 7:**
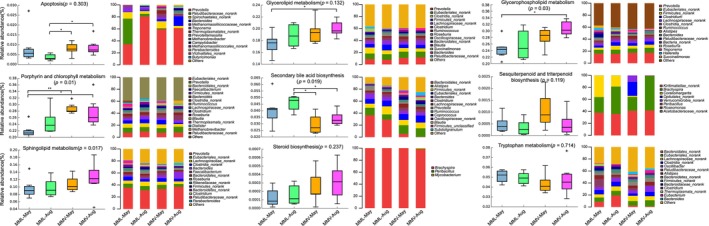
The relative abundance and bacterial source of the target KEGG pathways associated with altitude adaptation using 24 metagenomes. Pairwise comparisons were based on the Kruskal–Wallis test (Bonferroni *p*‐value). Aug, August; MML, *Macaca mulatta littoralis* living in the low‐altitude region; MMV, *Macaca mulatta vestita* living in the high‐altitude region.

Moreover, KEGG functional analysis using metagenomes indicated the role of the macaque gut microbiome in response to changes in altitude or altitude adaptation. The genes encoding the putative enzymes involved in porphyrin and chlorophyll metabolism were enriched in the high‐altitude samples, especially in May (Figures 6b and 7: KW test, *p* = 0.01). Another consensus feature in gut microbial function was that the genes encoding the putative enzymes involved in the sulfur relay system were significantly enriched in the high‐altitude samples (LEfSe analysis: Figure 6b). For example, in the sulfur relay system, ubiquitin and ubiquitin‐like proteins are signaling messengers that control many cellular functions, such as apoptosis and DNA repair (Herrmann et al., 2007; Raasi et al., 2001; Welchman et al., 2005). Interestingly, the genes encoding the putative enzymes involved in apoptosis and sphingolipid metabolism were enriched in the high‐altitude samples (Figure 7). High altitudes harsh environment results in apoptosis and DNA damage in living organisms (Kosanovic et al., 2019; Li et al., 2017; Møller et al., 2001; Seufferheld et al., 2008; Wu et al., 2020). Iron and sphingolipids are active players in (mal)adaptation to physiological hypoxia (Ottolenghi et al., 2020). Therefore, we deduced that (1) the stress of high‐altitude environments influences either the living animals or their symbiotic microbiota, and (2) the gut microbiota putatively responds to this harsh environment.

### 3.3 Spatial and temporal changes in the macaque gut microbial composition and function

The consistent spatial patterns of the fecal metabolomes between high‐ and low‐altitude samples in either May or August also reflect the potential dietary effects (Figure 2). For example, some metabolites belonging to flavonoids and carbohydrates were enriched in the low‐altitude samples (Figure 2a). Dietary flavonoids represent various polyphenolic compounds in plant foods, such as fruits, vegetables, grains, herbs, and beverages (Hooper et al., 2008). Fruits are rich in carbohydrates (Leopold et al., 2011). Here, we found that fruit intake was consistently higher in the low‐altitude macaques than in high‐altitude macaques (Table 1).

**TABLE 1:** Food contribution rate for the study group.

Month	Type of food	Food source	Contribution on rate (%)	Species number	Preferred family
May 2020 high altitude	Buds/Flower	Wild	12.26 ± 1.17	32	Ranunculaceae Rosaceae Asteraceae Cruciferae Pinaceae
	Young leaf	Wild	35.04 ± 4.35		
	Mature leaf	Wild	19.17 ± 2.78		
	Seed	Wild	10.29 ± 1.28		
	Petiole/Bark/Stem	Wild	15.36 ± 1.62		
	Other	Wild	7.88 ± 1.06		
August 2020 high altitude	Buds/Flower	Wild	11.58 ± 1.36	28	Ranunculaceae Rosaceae Asteraceae Cruciferae Pinaceae Leguminosae
	Young leaf	Wild	17.75 ± 2.72		
	Mature leaf	Wild	29.12 ± 4.46		
	Fruit/Seed	Wild	18.65 ± 3.15		
	Petiole/Bark/Stem	Wild	12.89 ± 1.32		
	Mushroom	Wild	2.64 ± 2.93		
	Other	Wild	7.37 ± 1.47		
May 2020 low altitude	Buds/Flower	Wild	20.45 ± 2.65	56	Fagaceae Rosaceae Cornaceae Moraceae Lauraceae Actinidiaceae
	Young leaf	Wild	32.67 ± 1.94		
	Mature leaf	Wild	28.63 ± 3.59		
	Fruit/Seed	Wild	11.52 ± 1.14		
	Other	Wild	6.73 ± 0.72		
August 2020 low altitude	Buds/Flower	Wild	5.20 ± 0.45	65	Fagaceae Rosaceae Cornaceae Moraceae Lauraceae Actinidiaceae
	Young leaf	Wild	8.72 ± 1.82		
	Mature leaf	Wild	35.87 ± 6.12		
	Fruit/Seed	Wild	46.38 ± 4.38		
	Mushroom	Wild	0.31 ± 0.12		
	Other	Wild	3.52 ± 0.51		

Moreover, primary bile acid biosynthesis was upregulated in the low‐altitude metabolomes (Figure 2c), and secondary bile acid biosynthesis was also consistently enriched in the low‐altitude gut metagenomes (Figure 7). Bile acids are synthesized in the liver in mammals (Hofmann et al., 2010; Russell, 2003). For example, in humans, the level of bile acid secretion into the intestine is 4–6 g per meal (Hofmann, 1999). Bile acid‐containing micelles aid lipases, which play roles in digesting and absorbing fat and nutrients from the diet (Hofmann & Borgström, 1964). In the intestine, primary bile acids (approximately 5% of the total) are often converted by gut bacteria to secondary bile acids (Ridlon et al., 2014). In our study, one unclassified genus from Bacteroidales played an essential role in secondary bile acid biosynthesis in the gut of the low‐altitude population (Figure 7). Thus, we speculated that the spatial difference in the metabolomes and gut microbiome between low‐ and high‐altitude macaques might be caused by the differences in the diets to some extent.

Furthermore, the high‐altitude samples showed significant enrichment of some metabolites belonging to the triterpenoid subclass, such as ganoderic acids. Triterpenes play essential roles in immune regulation and other biological activities in humans. Ganoderic acids (GAs) are critical bioactive constituents of the medicinal mushroom *Ganoderma lucidum* (Komoda et al., 1989; Xu et al., 2010). Some ganoderic acids have some biological activities, including hepatoprotective, antitumor, cardioprotective, and antioxidant (Cho et al., 2021; Gill & Kumar, 2019). Hypobaric hypoxia causes oxidative stress, and the body's antioxidant system plays a vital role in controlling it (Askew, 2002; Singh, 2022; Sinha et al., 2009). Ganoderma lucidum is a reddish laccate species of Ganoderma with a wild distribution in Europe and Asia (Zhou et al., 2015), and the study area in the high‐altitude region in the Tibetan Plateau is within one of the famous wild distribution regions of Ganoderma lucidum (Liu et al., 2021; Wang et al., 2015). Compared to that in the low‐altitude region, food sources for macaques living in the high‐altitude region are scarce. Thus, we speculated that macaques living at high altitudes seek different kinds of food, including Ganoderma, and the potential biological activities (e.g., antioxidation and cardioprotective roles) of the components of Ganoderma further drive macaque monkeys to choose these kinds of food.

**FIGURE 8:**
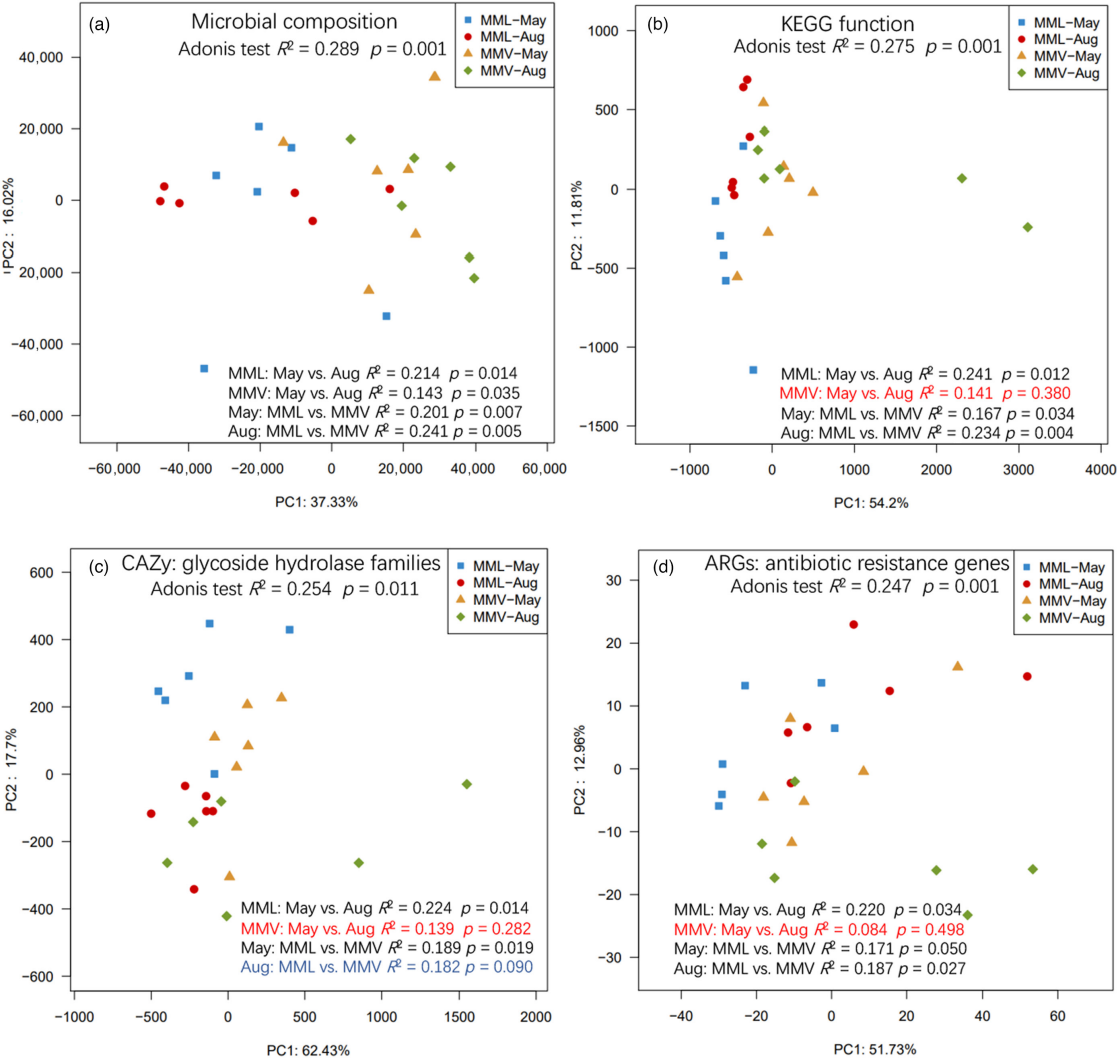
The PCoA ordination and adonis test (at 999 permutations with adjusted *p*‐value) based on the Bray–Curtis distance matrices in the macaque metagenomes based on (a) bacterial species; (b) KEGG function at the enzyme level; (c) CAZy glycoside hydrolase families; and (d) antibiotic resistance genes. Aug, August; MML, *Macaca mulatta littoralis* living in the low‐altitude region; MMV, *Macaca mulatta vestita* living in the high‐altitude region.

The metagenomic data display the seasonal dynamics of gut microbial function and the effects (Figure 8). Significant seasonal changes in microbial composition, KEGG function, glycoside hydrolase families, and ARGs were found in the low‐altitude macaques, but such changes in gut microbial function were not found in high‐altitude macaques. This finding might be attributed to differences in dietary composition in low‐altitude macaques. For example, the main component of the diet in May was young leaves (29%), while that in August was fruit/seed (46%) (Table 1). A seasonal diet drives seasonal changes in the gut microbial community in several primates (Amato et al., 2015; Baniel et al., 2021; Hicks et al., 2018; Li et al., 2023; Orkin et al., 2019; Rudolph et al., 2022). However, the seasonal change in the diet composition was not profound in high‐altitude populations, and the main dietary components were leaves and buds/flowers (Table 1), which might be partially attributed to the harsh environment and slow turnover rate of the forest. In August, we found no significant change in the glycoside hydrolase families involved in carbohydrate metabolism between low‐ and high‐altitude gut metagenomes. The increased fruit intake may have caused this during this season. Therefore, the spatial–temporal change in gut microbial function was more profound in the low‐altitude macaques than in the high‐altitude population.

## 4 CONCLUSION

Moreover, the tree species richness negatively correlates with altitude (Sinha et al., 2018), which might lead to a restriction in food availability for living mammals in high‐altitude regions. Here, based on the evidence from the fecal metabolome and gut microbiome, the harsh environment (e.g., physiological challenges and restricted food resources) might shape the macaque monkeys' gut microbial composition and function. However, whether the responded gut microbiome would further help the host in the local adaptation still need to be tested.

## FUNDING INFORMATION

Financial support was provided by the Second Tibetan Plateau Scientific Expedition and Research Program (2019QZKK0501), the Tibet Science and Technology Program (XZ202001ZY0012G), the Foundation of Key Laboratory of Southwest China Wildlife Resources Conservation (Ministry of Education) (XNYB22‐01), and high‐level talent start‐up funding in Nanjing University of Chinese Medicine.

## CONFLICT OF INTEREST STATEMENT

The authors declare no competing financial interests.

## Supporting information


Appendix S1

## Data Availability

The raw sequence data have been deposited in NCBI (PRJNA916292 and PRJNA916383).
