# Supplementary material for: The putatively high‐altitude adaptation of macaque monkeys: Evidence from the fecal metabolome and gut microbiome
Source: Evol Appl. 2023 Sep 25;16(10):1708–20. doi: 10.1111/eva.13595 (PMC10660799; doi:10.1111/eva.13595)
Supplement: Supplementary file 1 — Appendix S1 [file EVA-16-1708-s001.docx]

**Supporting Information**

**Metabolome protocol, SI Tables, and Figures**

**Metabolome protocol**

Fifty-nine fecal samples were used in the metabolome experiment. An accurately weighed 60 mg sample was transferred to a 1.5 mL Eppendorf tube. Two small steel balls were added to the tube. Twenty microliters of L-2-chlorophenylalanine (0.3 mg/mL) dissolved in methanol as an internal standard and 0.6 mL of a mixture of methanol and water (7/3, vol/vol) were added to each sample, and the samples were placed at -20 °C for 2 min. Then, the samples were ground at 60 Hz for 2 min, and the whole samples were extracted by ultrasonication for 30 min in an ice-water bath and placed at -20 °C for 20 min. The samples were centrifuged at 4 °C (13,000 rpm) for 10 min prior to decanting 150 μL supernatants from each tube, which were collected using crystal syringes, filtered through 0.22 μm microfilters and transferred to LC vials. The vials were stored at -80 °C until LC‒MS analysis. QC samples were prepared by mixing aliquots of all the samples to obtain a pooled sample.

An AB ExionLc system (AB SCIEX, Framingham, MA) coupled with an AB SCIEX Triple TOF 5600 System (AB SCIEX, Framingham, MA) was used to analyze the metabolic profiling in both ESI positive and ESI negative ion modes. An ACQUITY UPLC HSS T3 column (1.8 μm, 2.1 × 100 mm) was employed in both positive and negative modes. The binary gradient elution system consisted of (A) water (containing 0.1% formic acid, v/v) and (B) acetonitrile (containing 0.1% formic acid, v/v), and separation was achieved using the following gradient: 0 min, 5% B; 2 min, 5% B; 4 min, 25% B; 8 min, 50% B; 10 min, 80% B; 14 min, 100% B; 15 min, 100% B; 15.1 min, 5% and 16 min, 5% B. The flow rate was 0.35 mL/min, and the column temperature was 45 ℃. All the samples were kept at 4℃ during the analysis. The injection volume was 2 μL. Data acquisition was performed in full scan mode (m/z ranges from 100 to 1000) combined with IDA mode. The parameters for mass spectrometry were as follows: ion source temperature, 550 °C (+) and 550 °C (−); ion spray voltage, 5500 V (+) and 4500 V (−); curtain gas, 35 PSI; nebulizer gas, 55 PSI; auxiliary gas, 55 PSI; declustering potential, 80 V (+) and 80 V (−); collision energy, 10 eV (+) and 10 eV (−); and interface heater temperature, 550 °C (+) and 550 °C (−). For IDA analysis, the range of m/z was set as 40–1000, and the collision energy was 35 eV. The original LC‒MS data were processed by Progenesis QI V2.3 software (Nonlinear, Dynamics, Newcastle, UK) for baseline filtering, peak identification, integration, retention time correction, peak alignment, and normalization. The main parameters applied were as follows: 5 ppm precursor tolerance, 10 ppm product tolerance, and 5% product ion threshold. Compound identification was performed based on the precise mass-to-charge ratio (M/z), secondary fragments, and isotopic distribution using The Human Metabolome Database (HMDB) and Lipidmaps (V2.3), Metlin, EMDB, PMDB, and self-built databases to perform qualitative analysis. The extracted data were then further processed by removing any peaks with a missing value (ion intensity = 0) in more than 50% of the groups, by replacing the zero value by half of the minimum value, and by screening according to the qualitative results of the compound. Compounds with resulting scores below 36 (out of 60) points were also deemed to be inaccurately identified and removed. A data matrix was prepared by combining the positive and negative ion data.

**Table S1 The dataset in this study**

| **Species** | **16s ID** | **Meta ID** | **Metabolome ID** | **Sampling time** |
| --- | --- | --- | --- | --- |
| *M.m.littoralis 1* | JF1 | JF1 | JF1 | May |
| *M.m.littoralis 2* | JF2 |  | JF2 | May |
| *M.m.littoralis 3* | JF3 |  | JF3 | May |
| *M.m.littoralis 4* | JF4 | JF4 | JF4 | May |
| *M.m.littoralis 5* | JF5 | JF5 | JF5 | May |
| *M.m.littoralis 6* | JF6 |  | JF6 | May |
| *M.m.littoralis 7* | JF7 | JF7 | JF7 | May |
| *M.m.littoralis 8* | JF8 |  | JF8 | May |
| *M.m.littoralis 9* | JF9 |  | JF9 | May |
| *M.m.littoralis 10* | JF10 |  | JF10 | May |
| *M.m.littoralis 11* | JF11 |  | JF11 | May |
| *M.m.littoralis 12* | JF12 |  | JF12 | May |
| *M.m.littoralis 13* | JF13 | JF10 | JF13 | May |
| *M.m.littoralis 14* | JF14 |  | JF14 | May |
| *M.m.littoralis 15* | JF15 |  | JF15 | May |
| *M.m.littoralis 16* | JF16 | JF16 | JF16 | May |
| *M.m.littoralis 17* | JF17 |  | JF17 | May |
| *M.m.littoralis 18* | JF18 |  | JF18 | May |
| *M.m.littoralis 19* | JF19 |  | JF19 | May |
| *M.m.littoralis 20* | JF20 |  | JF20 | May |
| *M.m.littoralis 21* | FS1 | FS1 | FS1 | Aug |
| *M.m.littoralis 22* | FS2 |  | FS2 | Aug |
| *M.m.littoralis 23* | FS3 | FS3 | FS3 | Aug |
| *M.m.littoralis 24* | FS4 | FS4 | FS4 | Aug |
| *M.m.littoralis 25* | FS5 | FS5 | FS5 | Aug |
| *M.m.littoralis 26* | FS6 |  | FS6 | Aug |
| *M.m.littoralis 27* | FS7 |  | FS7 | Aug |
| *M.m.littoralis 28* | FS8 | FS8 | FS8 | Aug |
| *M.m.littoralis 29* | FS9 |  | FS9 | Aug |
| *M.m.littoralis 30* | FS10 | FS10 | FS10 | Aug |
| *M.m.vestita 1* | DG1 | DG1 | DG1 | May |
| *M.m.vestita 2* | DG2 |  | DG2 | May |
| *M.m.vestita 3* | DG3 | DG3 | DG3 | May |
| *M.m.vestita 4* | DG4 |  | DG4 | May |
| *M.m.vestita 5* | DG5 |  | DG5 | May |
| *M.m.vestita 6* | DG6 | DG6 | DG6 | May |
| *M.m.vestita 7* | DG7 |  | DG7 | May |
| *M.m.vestita 8* | DG8 | DG8 | DG8 | May |
| *M.m.vestita 9* | DG9 |  | DG9 | May |
| *M.m.vestita 10* | DG10 |  | DG10 | May |
| *M.m.vestita 11* | DG11 |  | DG11 | May |
| *M.m.vestita 12* | DG12 |  | DG12 | May |
| *M.m.vestita 13* | DG13 |  | DG13 | May |
| *M.m.vestita 14* | DG14 |  | DG14 | May |
| *M.m.vestita 15* | DG15 | DG15 | DG15 | May |
| *M.m.vestita 16* | DG16 |  | DG16 | May |
| *M.m.vestita 17* | DG17 |  | DG17 | May |
| *M.m.vestita 18* | DG18 | DG18 | DG18 | May |
| *M.m.vestita 19* | DG19 |  | DG19 | May |
| *M.m.vestita 20* | DA1 |  | DA1 | Aug |
| *M.m.vestita 21* | DA2 |  | DA2 | Aug |
| *M.m.vestita 22* | DA3 |  | DA3 | Aug |
| *M.m.vestita 23* | DA4 | DA4 | DA4 | Aug |
| *M.m.vestita 24* | DA5 | DA5 | DA5 | Aug |
| *M.m.vestita 25* | DA6 | DA6 | DA6 | Aug |
| *M.m.vestita 26* | DA7 | DA7 | DA7 | Aug |
| *M.m.vestita 27* | DA8 | DA8 | DA8 | Aug |
| *M.m.vestita 28* | DA9 | DA9 | DA9 | Aug |
| *M.m.vestita 29* | DA10 |  | DA10 | Aug |


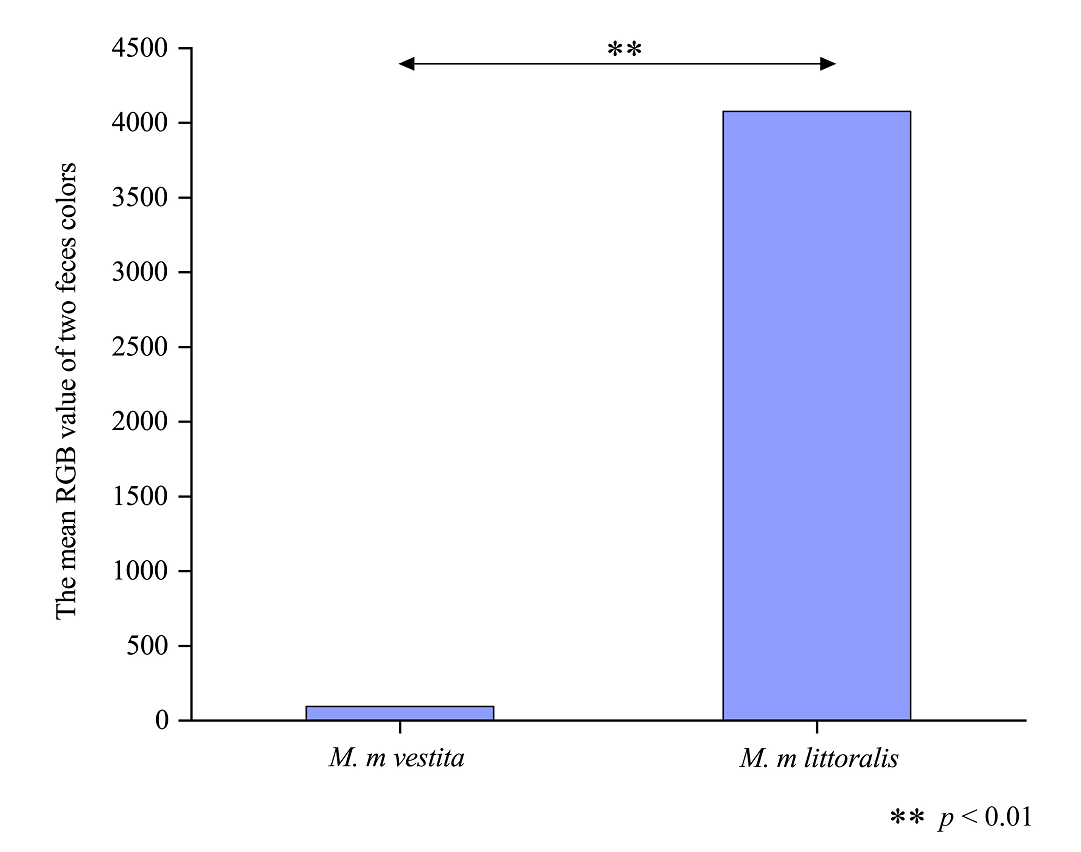


**Figure S1** **The color of the feces.** This analysis showed that the significant difference in the mean RGB (Red Green Blue color channel) values of two feces colors (Wilcoxon Test: Z=-2.934, *p*<0.01).

**Figure S2 The gut microbial composition among these macaque groups.** (A) The top 50 genus. Linear discriminant analysis effect size was used to determine the significant difference in relative abundance of gut microbes between low- and high-altitude macaque population in each sample season (B, May; C, August). MMV, *Macaca mulatta vestita* living in the high-altitude region. MML, *Macaca mulatta littoralis* living in the low-altitude region. Aug, August.
